# National Survey on Infection Prevention and Control in United States Emergency Departments

**DOI:** 10.5811/westjem.46582

**Published:** 2025-11-26

**Authors:** Laya Dasari, Molly L. Paras, Samantha L. Pellicane, Eileen F. Searle, Amy Courtney, Julio Ma Shum, Krislyn M. Boggs, Janice A. Espinola, Ashley F. Sullivan, Carlos A. Camargo, Jeremiah D. Schuur, Erica S. Shenoy, Paul D. Biddinger

**Affiliations:** * Massachusetts General Hospital, Center for Disaster Medicine, Department of Emergency Medicine, Boston, Massachusetts; † Massachusetts General Hospital, Division of Infectious Diseases, Boston, Massachusetts; ‡ Harvard Medical School, Boston, Massachusetts; § Infection Control, Mass General Brigham, Boston, Massachusetts; ¶ Lawrence General Hospital, Lawrence, Massachusetts; || Emergency Medicine Network, Department of Emergency Medicine, Massachusetts General Hospital, Boston, Massachusetts; # Massachusetts General Hospital, Department of Emergency Medicine, Boston, Massachusetts; ** Tufts University School of Medicine, Boston, Massachusetts

## Abstract

**Introduction:**

In the emergency care setting, implementation of infection prevention and control (IPC) practices can be challenging due to numerous factors including emergency department (ED) crowding and boarding of patients, high staff-turnover rates, and acuity of patient needs. Understanding how the unique nature of the ED environment impacts IPC implementation is essential to reducing healthcare-associated infections and to improving patient safety. In this study we aimed to assess ED leaders’ perceptions of IPC practices to identify areas for potential intervention and inform targeted process improvement initiatives.

**Methods:**

Between January–July 2023, ED leaders across the United States were queried about their IPC practices using the National Emergency Department Inventories (NEDI)-USA survey, which is administered annually to all EDs in the US. An expanded survey was administered in a subset of EDs to assess healthcare personnel training for IPC, reported adherence to recommended practices and policies related to disinfection of reusable medical equipment and environment, use of personal protective equipment, hand hygiene practices, patient care space cleaning and disinfection, use of transmission-based precautions signage, risk perceptions of how healthcare personnel practice contributes to healthcare-associated infections and barriers to appropriate room cleaning.

**Results:**

Of the 289 facilities surveyed, 159 (55%) responded, and among responding EDs, 67 (42%) reported seeing ≥ 40,000 patients in the prior year. Regarding healthcare personnel training, 84% (131/156) of ED leaders reported that ≥80% of their ED healthcare personnel were correctly trained in IPC procedures according to their hospital’s policies. Perception of healthcare personnel compliance with IPC practices, however, was lower. Although 75% (118/157) of EDs reported > 80% compliance with correct N95 respirator use, compliance with transmission-based precaution signage was identified as a significant gap, with 30% (47/159) of EDs reporting that they never, rarely, or only sometimes posted signs for patients who required them. Further, 69% (61/89) of EDs reported that they never, rarely, or only sometimes posted transmission-based precaution signs for patients in hallways or overflow treatment spaces.

**Conclusion:**

This national survey found that ED leaders perceive that their healthcare personnel have a high level of knowledge of IPC policies and compliance with some, but not all, IPC policies in the ED. The overall high perceptions of compliance stand in contrast to prior published observations of poor IPC practice in ED settings, suggesting complex relationships between perception and practice that may impact patient safety outcomes. These findings can guide future targeted interventions to improve IPC compliance, reduce healthcare-associated infections, and improve patient safety in emergency settings.

## INTRODUCTION

The emergency department (ED) is often seen as the frontline of healthcare delivery, characterized by an unpredictable and dynamic environment that can amplify challenges seen throughout the healthcare system.^1^ While infection prevention and control (IPC) practices are critical for maintaining patient safety in all healthcare settings, successfully adhering to them in the ED environment presents unique challenges that stem from multiple factors including ED crowding, patients presenting with a diverse array of acute and often undifferentiated medical conditions, and comparatively high rates of staff turnover.^2^–^8^ The urgent nature of providing clinical care in the ED can negatively affect healthcare personnel ability to implement essential IPC measures, such as proper hand hygiene, appropriate use of personal protective equipment, and thorough cleaning and disinfection of spaces and equipment between patient encounters.^9^–^11^ These challenges in maintaining effective IPC practice increase the risk of healthcare-associated infections and the potential for iatrogenic morbidity and mortality.^12^,^13^ Healthcare-associated infections are a major public health concern, causing an estimated 75,000 deaths annually in the United States. Advancing IPC efforts in emergency care is both timely and necessary.^12^

Although IPC is a critical component of safe care delivery, previous research has shown that compliance remains persistently low. Reported hand hygiene adherence rates range from 20–70%, frequently lower than in other hospital units such as intensive care units or inpatient wards.^15^,^16^ Barriers commonly identified in those settings, such as time constraints, staffing limitations, and environmental challenges, are often even more pronounced in the ED.^17^,^18^ Yet despite these barriers, few studies have specifically assessed IPC practices in emergency settings, highlighting a persistent gap in the literature. To address this gap, we sought to evaluate ED leaders’ perceptions of the current state of IPC practices within US EDs, focusing on reported compliance, barriers, and perceptions of risk. Our study included select items from a 2011 IPC survey to facilitate longitudinal comparisons, and the survey was administered to the same cohort of responding hospitals.^19^

## METHODS

### Study Design

This was a cross-sectional survey of US EDs. The Mass General Brigham Institutional Review Board reviewed this project and classified it as exempt (IRB Protocol: 2005P000015). This study was reported in accordance with the STROBE (Strengthening the Reporting of Observational Studies in Epidemiology) guidelines.^20^

### Sample

We selected EDs to receive the survey using the National ED Inventory (NEDI)-USA, a database of all non-federal, non-specialty EDs open 24 hours per day, seven days per week, and 365 days per year.^19^ The NEDI-USA dataset also includes publicly available information about Urban Influence Codes developed by the the Department of Agriculture Economic Research Service (to classify EDs as urban, large rural, or small rural), academic ED and hospital status, Council of Teaching Hospital status, and Critical Access Hospital status.^21^–^24^

**Population Health Research Capsule**

What do we already know about this issue?

*Infection prevention and control (IPC) compliance in emergency departments is low, with adherence rates frequently below those in other hospital settings.*

What was the research question?

*How do US emergency department leaders perceive training, compliance, and barriers to IPC practices?*

What was the major finding of the study?

*Of 159 EDs, 84% reported high rates of IPC staff training, and 71% reported high rates of hand hygiene compliance.*

How does this improve population health?

*Gaps between perceptions and realities from the literature in ED IPC point to opportunities to improve IPC compliance and patient safety.*

The selected EDs previously participated in a survey assessing IPC characteristics in 2011.^19^ The original sample was selected using multistage stratification, including purposeful oversampling of high-volume EDs (those with > 50,000 annual visits) and teaching hospitals. These facilities tend to have a higher burden of healthcare-associated infections and exert greater influence on the practice of emergency medicine, making them particularly relevant for tracking changes in IPC implementation across the healthcare system.^25^ Of the 301 EDs that responded to the earlier survey, 12 had closed by 2022 and thus were excluded. The remaining 289 eligible EDs were in each of the four US regions (Northeast, Midwest, South, and West) in 48 states plus the District of Columbia. All EDs were hospital-based; none were freestanding. Within each ED, key leadership personnel targeted for survey response included physician medical directors, nurse managers, infection control leaders, and department administrators. We selected these roles based on their decision-making roles in clinical operations and infection prevention.

### Survey and Administration

The survey instrument was developed by the research team, including subject matter experts in IPC, ED operations, and research design and was built upon the previous 2011 survey.^19^ The present study included select items from the 2011 study of the same ED cohort to enable longitudinal comparison, while expanding into new areas of inquiry. Between January–July 2023, a three-page survey (Supplement A) was administered to study participants to characterize emergency care and ED IPC policies and practices in 2022. The survey included questions about ED characteristics (eg, annual visit volume). The IPC policies and practices were assessed with questions related to healthcare personnel training, reported adherence to IPC policies related to disinfection of reusable medical equipment and environment, use of personal protective equipment, hand hygiene practice, care-space cleaning and disinfection, use of transmission-based precautions signage, risk perceptions of how ED practice contributes to healthcare-associated infections, and barriers to appropriate room cleaning. Except for hand hygiene compliance, for which respondents were asked to report based on available audit data, all survey responses reflected ED leadership’s self-reported assessments rather than verified metrics. All questions about IPC asked the respondents to select from multiple options, with the exception of one ranking question. A note on a question modification is available (Supplement B).

We administered the survey using mixed modalities. It was sent by US mail to ED leaders up to three times between January–April 2023. A link to an online version of the survey was included in each mailing. Emergency department leaders who provided an email address in response to a previous NEDI-USA survey also received a copy of the survey by email. Those who returned an incomplete survey were contacted by phone or email to re-administer questions left blank. We contacted EDs that did not respond to mailed surveys by telephone for survey completion.

### Data Analysis

We calculated response rate by using the number of EDs that responded divided by the total number of potential respondent EDs, all of which were confirmed eligible before being surveyed.^26^,^27^ Data were summarized with descriptive statistics (eg, counts, proportions). For ease of interpretation, we grouped survey response options estimating ED compliance rates into two categories: low/medium (0–79%); and high (≥ 80%). This threshold (≥ 80%) was selected as it represented the highest response category for many survey items and is in alignment with previous research. It was then applied uniformly across all variables to facilitate consistent analysis. This cutoff is also consistent with thresholds used in prior IPC studies, where ≥ 80% has commonly been used to indicate high compliance.^28^,^29^ For all survey items, we excluded EDs that did not provide a response (≤ 3% per item). Bivariate comparisons of categorical variables were made using χ2 tests. A two-tailed *P* < .05 was considered statistically significant. We completed analyses in Stata 18.0 (StataCorp, LLC, College Station, TX).^30^

## RESULTS

Of the 289 EDs surveyed, 159 (55%) responded (Table 1). Comparing the characteristics of responding vs nonresponding EDs, there was no material difference by US region, academic ED status, and Council of Teaching Hospitals status. However, responding EDs more often had annual visit volumes of < 15,000 visits per year (31% vs 16%; *P* = .01), were in rural areas (31% vs 19%; *P* = .05), and designated as a Critical Access Hospital (24% vs 11%; *P* < .01). Among responding EDs, the 2022 ED visit volume remained at a comparable level, with about a third (46, 29%) of EDs having < 15,000 visits per year.

**Figure 1:**
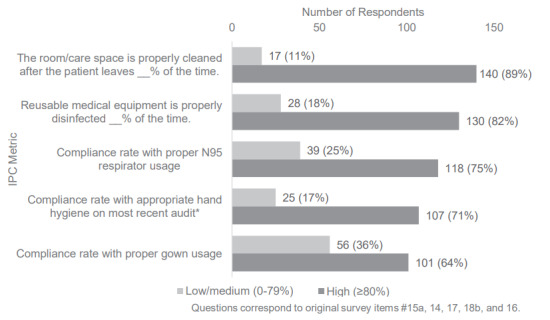
Reported compliance across infection prevention domains. Questions were edited for brevity, and clarifications were provided on the survey of US emergency department infection prevention and control compliance. The room/care space cleaning was specified to hospital’s standard. Reusable medical equipment disinfection was specified according to hospital’s policies. Compliance with N95 respirator usage specified proper donning and doffing, ensuring a clean-shaven face, and use when specified when indicated by hospital policy. Donning and doffing PPE was described in the proper gown usage question. *18 emergency departments (ED) indicated “unsure” of hand hygiene audit compliance (not shown); 9 EDs did not audit hand hygiene compliance and were excluded. *PPE*, personal protective equipment; *US*, United States.

Regarding healthcare personnel training, 84% (131/156) of ED leaders reported that ≥ 80% of their ED staff are correctly trained in their hospital’s IPC policies and procedures. In addition, compliance with IPC policies was reported as relatively high in some metrics, with 89% (140/157) of EDs reporting ≥ 80% compliance with appropriate room cleaning and disinfection after the patient leaves, and 82% (130/158) of EDs reporting ≥ 80% compliance with policies regarding cleaning reusable medical equipment (Figure 1). Hand hygiene compliance in EDs that reported conducting auditing was relatively low compared to the other domains, with only 71% (107/150) of respondents reporting ≥ 80% compliance with recommended hand hygiene protocols. Proper gown usage was among the lowest reported compliance with 64% (101/157) reporting ≥ 80% compliance.

**Figure 2:**
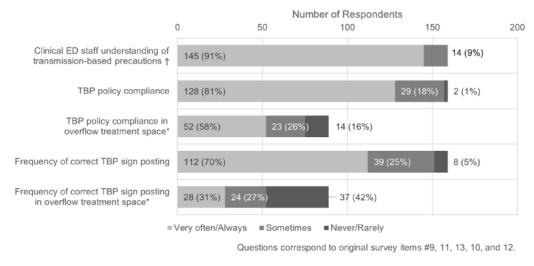
Transmission-based precaution understanding and adherence. In a study of US emergency department (ED) infection prevention and control compliance, ED leaders were asked, “How well do you believe your clinical ED staff understand the concept of transmission-based precautions?” with the examples of contact, droplet, and airborne provided. Overflow treatment spaces include hallway spaces. Questions and responses were edited for brevity. † Clinical ED staff understanding was asked on a scale “Very well” to “Not at all.” Answers are pooled “Very well/Well” and “Somewhat.” No EDs answered “Not well/Not at all.” * 69 EDs indicated that use of overflow treatment space was not applicable and were excluded. *TBP*, transmission-based precautions.

Approximately 91% (145/159) of respondents reported that their healthcare personnel have a sufficient understanding of transmission-based precautions (Figure 2); however, 19% (31/159) indicated that transmission-based precaution policies are never, rarely, or sometimes followed. Compliance with signage was also identified as a significant gap, with 30% (47/159) of EDs reporting that they never, rarely, or only sometimes posted signs for patients who required them. This gap was more pronounced with patients placed in hallways or overflow treatment spaces. In hospitals that use hallway or overflow spaces, 42% (37/89) reported that transmission-based precautions were followed correctly never, rarely, or sometimes, and 69% (61/89) of EDs never, rarely, or only sometimes posted transmission-based precaution signs.

**Figure 3:**
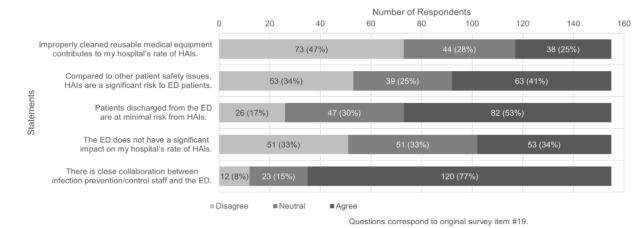
Perceptions of importance of infection prevention in the emergency department (ED). In a study on US ED infection prevention and control compliance, ED leaders were asked to indicate their level of agreement with each of the statements. *HAI*, healthcare-associated infection.

Leaders’ perceptions of the overall importance of IPC policy and procedure adherence varied significantly (Figure 3). Most ED leaders (59%, 92/155) reported that they did not perceive healthcare-associated infections to pose a significant risk to patients relative to other patient safety issues in the ED or felt neutral about the issue. Only about one-third of respondents (34%, 53/155) felt that their ED did not have a significant impact on risk of healthcare-associated infections; however, approximately 77% of respondents (120/155) perceived a strong collaboration between IPC staff and the ED.

**Figure 4:**
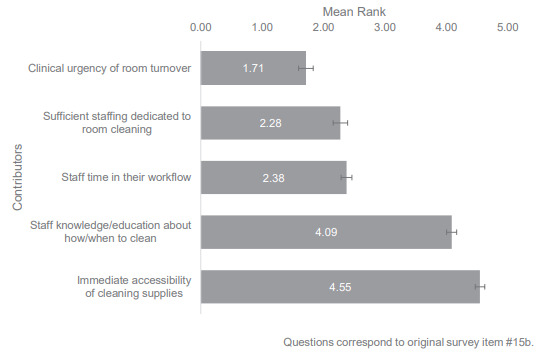
Rankings of perceived barriers to proper room cleaning. In a study of infection prevention and control compliance in US emergency departments, participants were asked to “rank from 1 to 6, with 1 being the biggest contributor and 6 being the smallest contributor, the reasons you believe the room/care space is not properly cleaned to your hospital’s standard.” Lower mean rank indicates barrier perceived as more significant contributor to inadequate room cleaning. Emergency department leaders were also able to write in “other” responses, which were not included in the mean rank calculation. Repeated numbers were not allowed, and the mean rank is reported. Standard error was used for error bars. “Other” responses not included in calculation: “accidental misses”; “didn’t realize”; room/equipment out of service; language barrier with cleaning staff; and “lack of integrity.”

About half of EDs (49%, 77/157) reported that a room/care space is always cleaned to their hospital’s standard after a patient leaves, and the 80 others were asked to rank factors that affect the ED’s ability to appropriately clean rooms between patient care episodes (Figure 4). Respondents identified clinical urgency of room turnover as the main negative influence, with a mean rank of 1.71. Other important factors reported included having sufficient staffing dedicated to room cleaning and sufficient time available in staff workflows, with mean ranks of about 2.3 and 2.4, respectively. Considered less influential were factors such as staff knowledge and education about cleaning procedures, with an approximate mean rank of 4.1, and the immediate accessibility of cleaning supplies, with a mean rank of about 4.6.

### Comparison to 2011 Survey Results

Of the 412 EDs surveyed in 2011, 301 (73%) responded. Regarding questions that were consistent across both surveys, more EDs reported at least 80% correct hand hygiene upon audit in 2022 vs 2011 (71% vs 46%, respectively). Respondents were less likely to agree that healthcare-associated infections were a significant risk to patients compared with other safety concerns (41% vs 63%) and more likely to believe that patients discharged from the ED are at minimal risk of healthcare-associated infections (53% vs 31%) in 2022 vs 2011. Further, fewer ED leaders felt that the ED does not have a significant impact on risk of healthcare-associated infections (34% vs 23%) and more respondents perceived a strong collaboration between IPC staff and the ED in 2022 vs 2011 (77% vs 63%).

## DISCUSSION

In this national survey we aimed to evaluate the reported compliance with IPC policies in EDs across various US hospitals, identifying strengths and gaps in IPC adherence as perceived by ED leaders. In this study, ED leaders perceived their healthcare personnel to have a high level of knowledge about IPC policies, and leaders reported generally high compliance across most IPC domains including disinfection of reusable medical equipment, hand hygiene, and room/care space cleaning and disinfection. This is generally consistent with previous studies that have shown that healthcare personnel, including those in EDs, self-report high levels of knowledge of recommended IPC practices.^31^,^32^ Additionally, a recent meta-analysis found that healthcare personnel generally demonstrate adequate to high levels of knowledge concerning standard precautions, hand hygiene, and care pertaining to specific diseases.^33^

However, the finding that ED leaders perceived a generally high level of compliance contrasts with the broader observational studies in the literature, which typically indicate low compliance in ED settings.^34^–^36^ For example, a study that examined the terminal cleaning thoroughness of 23 acute care hospitals found that only 49% of surfaces were sufficiently cleaned and disinfected, significantly lower than most EDs reported in this study. 37 Similarly, even after intervention, an observational study reported compliance with recommended hand hygiene policies to be 44.9%—again significantly lower than most respondents reported in this study.^38^ This discrepancy suggests potential differences between leadership perceptions and the realities captured by direct observations.

This divergence in perceived vs observed IPC practice has meaningful clinical implications. Healthcare-associated infections are associated with considerable morbidity and mortality, contributing to an estimated 75,000 deaths annually in US hospitals.^14^ While standard surveillance programs are designed to attribute healthcare-associated infections to inpatient settings, there is growing recognition that breakdowns in infection prevention practices in the ED do contribute.

Interestingly, we also found that most ED leaders do not perceive healthcare-associated infections as a significant risk relative to other patient safety issues, with only a third believing the ED significantly impacts hospital-associated infection rates. While previous research has shown that ED leadership recognizes the value of specific infection prevention guidelines, broader perceptions of the risk of healthcare-associated infections have not been well-studied.^39^ Although direct attribution of healthcare-associated infections to ED care is challenging due to existing standardized surveillance definitions, evidence suggests that ED practices, such as unnecessary urinary catheter placement, contribute to catheter-associated urinary tract infections.^40^ This potential impact, combined with the ED’s role as a primary entry point for patients with diverse infectious conditions requiring specific precautions, suggests a possible disconnect between ED leaders’ risk perception and the department’s role in IPC, patient safety, and quality of care.

This study also documents IPC challenges that are specific to the ED environment, such as adherence to policy in non-traditional care spaces like hallways and overflow treatment spaces. In these areas, which have been increasingly used in EDs across the US, compliance with transmission-based precautions was reported to be significantly lower as compared to care delivered in typical care areas.^41^ The most significant barriers to proper room cleaning and disinfection between patients identified in the survey—clinical urgency of room turnover, staffing constraints, and time limitations—also align with previous findings.^2^,^42^,^43^ Studies have documented that time pressure for rapid room turnover contributes to incomplete surface disinfection, particularly of high-touch surfaces in ED treatment areas.^44^ These reported challenges are consistent with previous studies that have noted how ED conditions, including urgency, crowding, and hallway care delivery, often compete with IPC protocols.^11^,^36^,^45^

Importantly, these strategies, including care in overflow areas, while aimed at addressing crowding and throughput, may inadvertently compromise IPC adherence and increase patient risk.^46^ These adaptations have become increasingly common, but their downstream consequences have been under-investigated and are not routinely reported; even local IPC lapses in the ED may contribute to infection risk across the broader continuum of care.

Comparison with the 2011 survey data reveals that ED leaders in 2022 reported higher levels of hand hygiene compliance and perceived stronger collaboration with IPC staff. Fewer leaders now view healthcare-associated infections as a significant patient safety risk or see the ED as a key contributor to those infections. While these shifts could reflect increased confidence in ED practices or the emergence of competing safety concerns, such as crowding or violence, they may also suggest a potential underestimation of the ED’s role in infection risk.^47^ This is particularly noteworthy given the ongoing discrepancy between reported and observed adherence. If perceived improvements outpace behavioral change, the ED’s contribution to preventable healthcare-associated infections may remain underestimated and not addressed.

## LIMITATIONS

Several limitations must be acknowledged in interpreting the results of this study, particularly regarding the potential for misestimation of practice adherence. While hand hygiene compliance rates were requested in the survey to be reported from audits, all other data in this study reflect ED leaders’ reported assessments of their department’s practices rather than verified observations or formal audit data. The approach of querying leaders, while valuable in understanding their perspectives, may not fully capture the reality of day-to-day IPC practices.

The discrepancy between reported and observational compliance rates in IPC in the literature suggests these findings may overestimate actual practice adherence. While ED leaders reported that they believed their EDs had medium to high rates of compliance with IPC policies in several areas, at the same time, they also described significant practice challenges such as inadequate IPC signage and inconsistent use of transmission-based precautions, especially when care was delivered in hallways or other surge spaces. Given that prior observational studies have consistently documented significant lapses in even basic IPC protocols, these reported challenges likely represent minimum estimates of actual practice gaps.

Multiple factors may have influenced the accuracy of leadership assessments. Social desirability bias may have affected responses, as ED leaders may be reluctant to document compliance with recommended IPCs lower than required, given that this affects patient safety and is a regulatory requirement of agencies such as The Joint Commission and is monitored by the Centers for Medicare & Medicaid Services.^48^,^49^ The proximity of the COVID-19 pandemic at the time of data collection may have further heightened this bias, as IPC practices had received increased attention and scrutiny across healthcare settings. Assessments by ED leaders may also be influenced by factors such as their specific role, level of involvement in daily operations, institutional reporting structures, and individual understanding of IPC best practices. While many ED leaders are involved in operations such as patient flow and clinical care, some IPC practices can often be the responsibility of personnel from separate departments, such as environmental services. This limitation is relevant for many metrics in this study, including data on environmental cleaning and transmission-based precautions, where compliance was assessed through leadership perception rather than audit data.

Even hand hygiene compliance rates, while derived from audits, may be subject to the Hawthorne effect and other observational biases. Additionally, because the survey did not ask how institutions assess IPC compliance across all metrics, variation in measurement approaches (eg, direct observation, electronic monitoring, or internal reporting) may further affect the consistency and comparability of reported adherence rates. These limitations underscore the need for future research that combines leadership perspectives with direct observational studies and input from frontline healthcare personnel to provide a more comprehensive picture of IPC realities in the ED.

Additionally, institutions with lower annual visit volumes and located in rural settings were over-represented in our study. This non-response bias may affect the overall representativeness of the findings, potentially influencing the reported compliance rates, given the different operational challenges faced by high-volume EDs. The over-representation of lower volume, rural EDs may have resulted in high reported compliance rates. Future studies may benefit from using shorter surveys, wave analysis, or modest incentives to improve response rates from under-represented institutions.^26^

### Implications for Future Work

The discrepancy between perceived and documented compliance highlights the importance of objective measurement. Individual institutions could benefit from monitoring systems to understand their specific IPC adherence rates, thereby helping ED leaders align their perceptions with operational realities. Given observational studies in the literature documenting IPC compliance challenges in EDs, we encourage future research on developing interventions that address systemic factors identified in this study such as time constraints, staffing levels, and the use of non-traditional care spaces. Such research would provide ED leaders with evidence-based options for improving compliance while accounting for the unique pressures and constraints of emergency care, as well as adding further evidence regarding the harms of ED crowding and hallway care and providing further impetus to urgently address these issues.

## CONCLUSION

Although 84% of ED leaders reported that the vast majority of staff were trained in infection prevention and control procedures, self-reported compliance varied considerably. High compliance was noted for room cleaning (89%), N95 use (75%), and hand hygiene (71%), but lower for gown use (64%). Signage for transmission-based precautions was particularly limited, with only 31% of EDs consistently posting signs in hallway or overflow spaces. These findings reflect substantial gaps even in self-reported data and likely underestimate true noncompliance, given prior observational studies. Without standardized measurement and ED-specific interventions, especially in non-traditional care areas, these risks to patients and staff are likely to persist.

## Supplementary Information





## Figures and Tables

**Table:** Hospital emergency department (ED) characteristics by survey responders vs non-responders. Chi-square test was used to test the association between ED characteristics and response (responding vs non-responding) to a national survey of US ED infection prevention and control compliance.

Characteristics	All US EDs (N = 5,580) n (%)	Responders (n = 159) n (%)	Non-responders (n = 130) n (%)	P-value
2021 ED Visit Volume				.01
< 15,000	2,410 (43)	49 (31)	21 (16)	
15,000–39,999	1,868 (33)	40 (25)	37 (28)	
≥ 40,000	1,302 (23)	70 (44)	72 (55)	
Region				.19
Northeast	628 (11)	27 (17)	35 (27)	
Midwest	1,513 (27)	43 (27)	34 (26)	
South	2,398 (43)	59 (37)	43 (33)	
West	1,041 (19)	30 (19)	18 (14)	
Setting				.05
Urban	3,705 (66)	109 (69)	105 (81)	
Large rural	761 (14)	23 (14)	14 (11)	
Small rural	1,114 (20)	27 (17)	11 (8)	
Academic ED	235 (4)	24 (15)	31 (24)	.06
Council of Teaching Hospital	261 (5)	36 (23)	35 (27)	.40
Critical Access Hospital	1,351 (24)	38 (24)	14 (11)	< .01

*US*, United States.
