# Supplementary material for: National Survey on Infection Prevention and Control in United States Emergency Departments
Source: West J Emerg Med. 2025 Nov 26;26(6):1781–9. doi: 10.5811/westjem.46582 (PMC12698160; doi:10.5811/westjem.46582)

MASSACHUSETTS GENERAL HOSPITAL
CENTER FOR DISASTER MEDICINE

National Emergency Department Inventory

EMNet

Dear Colleague:

We would like your help with a brief survey of 289 U.S. emergency departments (EDs). This is a follow-up to a similar study your ED participated in during 2011, as well as annual national surveys conducted over the past seven years. Your ED's responses to prior surveys have contributed to publications on several topics, including pediatric emergency care and ED telehealth use. If you would like a copy of any of these papers, please let us know and we'll send it to you!

This survey will gather information from ED leaders on 1) basic ED characteristics, and 2) views and activities regarding infection prevention and control in the ED. Your response to this survey will be used to perform health services research and to inform ongoing efforts to improve the accessibility, quality, and safety of care in the U.S. This study is funded in part by the Centers for Disease Control and Prevention.

Please answer questions for ____ that apply to the year **2022** (either fiscal or calendar year is acceptable). If you are unable to quickly find the precise answer, we welcome your best estimate. The survey takes approximately 15-20 minutes to complete.

Responses to questions #1-5 will be incorporated individually into the *findERnow* smartphone app. Responses to all other questions will not; those individual responses will be available only to research staff. Summaries of results will use aggregate responses only.

Without your response, this study cannot succeed. We hope that you will help us by completing this survey or asking a knowledgeable colleague for help. Please take a moment to send your answers to us in the attached, **pre-paid** envelope. If you prefer, we also welcome your responses:

- Online at https://redcap.link/CDC-ED2022 (URL is case sensitive)
- By email to emnet@partners.org
- By fax to 617-724-4050

If you have any questions, comments, or suggestions about the study, please contact us at emnet@partners.org or at 617-724-4069.

Thank you in advance for your assistance.

Best wishes,

Paul Biddinger, MD
Center for Disaster Medicine
Department of Emergency Medicine
Massachusetts General Hospital
Boston, MA

Carlos Camargo, MD, DrPH
Emergency Medicine Network (EMNet)
Department of Emergency Medicine
Massachusetts General Hospital
Boston, MA

SITE #

Please answer questions with estimates for the year **2022** (unless otherwise indicated)

BASIC ED CHARACTERISTICS

1) In 2022, was your ED open: a. 24 hours/day, 7 days/week? ◯ YES ◯ NO **b. 365 days/year ?** ◯ YES ◯ NO

If **NO** to either question, please explain: ______________________________

2) Please indicate the total number of patient visits at your ED and the 12-month reporting period to which they apply:

ED VISITS [ ][ ][ ][ ][ ][ ]

Reporting period: From ___/___ to ___/___
MM YY MM YY

3) Please indicate the approximate number of ED visits by children (e.g., age <18 years)*

CHILD ED VISITS [ ][ ][ ][ ][ ][ ]

Specify age cut-off if not <18 years: Age < _____ years

4) For EDs that regularly treat adults: **Does your ED have a dedicated area for children only (e.g., dedicated beds)?***

◯ YES ◯ NO ◯ Not applicable (e.g., children's hospital)

5) Do you have an identified coordinator(s) for pediatric emergency care in your ED?*

◯ YES ➜
◯ NO

5a. How many identified coordinators does your ED have? ◯ 1 ◯ 2 ◯ ≥ 3

5b. Please specify the type of coordinator(s): *(check all that apply)*

☐ Physician coordinator(s) ☐ Nurse coordinator(s) ☐ Other (e.g., PA, admin):__________

5c. How many total hours (weekly) do/does your identified coordinator(s) devote to the role?

◯ <5 hours ◯ 5 - 9.99 hours ◯ 10 - 19.99 hours ◯ 20 - 39.99 hours ◯ ≥ 40 hours

6) Does your ED *receive* telehealth services for patient evaluation from <u>another facility</u> in your health system or from an <u>outside organization</u>?*

◯ YES ➜
◯ NO

Does your ED utilize telehealth for: *(check all that apply)*

☐ Pediatrics ☐ Psychiatry ☐ Dermatology ☐ Transfer coordination ☐ COVID-19
☐ Stroke/neuro ☐ Trauma ☐ Radiology ☐ Disaster preparedness ☐ Other:__________

7) Does your hospital/ED *provide* telehealth services *out* for the evaluation of <u>patients in other EDs</u>?*

◯ YES ◯ NO ◯ Not sure

* additional info on the back of this booklet

8) Is at least one <u>attending physician</u> (not resident) on duty <u>in the ED</u> 24 hours/day, 7 days/week? *(Exclude on-call physicians).*

◯ YES
◯ NO ➜

If NO, when a <u>physician is not on duty in the ED</u>, is <u>any</u> physician available to the ED by two-way voice communication 24 hours/day, 7 days/week:

8a. From within your hospital? ◯ YES ◯ NO ◯ Not applicable (e.g., freestanding ED)
8b. From outside of your hospital? ◯ YES ◯ NO ◯ Not applicable (e.g., freestanding ED)

INFECTION CONTROL

9) In your experience, how well do you believe your clinical ED staff understand the concept of transmission-based precautions (e.g., contact, droplet, airborne)?

◯ Not at all ◯ Not well ◯ Somewhat ◯ Well ◯ Very well

10) In your experience, how often are transmission-based isolation precaution signs posted for each patient who requires them in your ED?

◯ Never ◯ Rarely ◯ Sometimes ◯ Very often ◯ Always

11) In your experience, how often are the hospital and departmental policies for transmission-based precautions in the ED correctly followed?

◯ Never ◯ Rarely ◯ Sometimes ◯ Very often ◯ Always

12) In your experience, for patients requiring transmission-based isolation precautions and having been placed in the hallway or other overflow treatment spaces in the ED, how often are transmission-based isolation precaution signs posted?

○ Never ○ Rarely ○ Sometimes ○ Very often ○ Always ○ Not applicable

13) In your experience, how often are the hospital and departmental policies for transmission-based precautions followed correctly for patients who are receiving care in hallways or overflow treatment spaces in the ED?

○ Never ○ Rarely ○ Sometimes ○ Very often ○ Always ○ Not applicable

14) Reusable medical equipment within the ED is properly disinfected (according to your hospital's policies) ___% of the time.

○ 0-19% ○ 20-39% ○ 40-59% ○ 60-79% ○ 80-89% ○ ≥90%

15) a. What percentage of the time is a room/care space cleaned to your hospital's standard after the patient leaves? ○ Never ○ 1-19% ○ 20-39% ○ 40-59% ○ 60-79% ○ 80-99% ○ Always *(skip to #16)*

b. Please rank from 1 to 6, with 1 being the biggest contributor and 6 being the smallest contributor, the reasons you believe the room/care space is not properly cleaned to your hospital's standard.

____Staff time in their workflow

____Immediate accessibility of cleaning supplies

____Clinical urgency of room turnover (i.e., need to get the next patient in the room before full cleaning)

____Staff knowledge/education about how/when to clean

____Sufficient staffing dedicated to room cleaning

____Other (please indicate: ____)

16) Please estimate the compliance rate for ED staff with proper gown usage (donning and doffing) when indicated by your hospital policy.

○ 0-19% ○ 20-39% ○ 40-59% ○ 60-79% ○ ≥80%

17) Please estimate the compliance rate for ED staff with proper N95 respirator usage (donning and doffing, clean-shaven face, etc.) when indicated by your hospital policy.

○ 0-19% ○ 20-39% ○ 40-59% ○ 60-79% ○ ≥80%

18) Is ED staff compliance with hand hygiene audited?

○ No *(skip to #19)* ○ Yes, by direct observation ○ Yes, by other measures

a. What was the compliance with appropriate hand hygiene for your ED on the most recent audit?

○ 0-19% correct hygiene ○ 40-59% correct hygiene ○ 80% or more correct hygiene
○ 20-39% correct hygiene ○ 60-79% correct hygiene ○ Unsure

19) Please answer if you agree or disagree with each of the following statements by checking the appropriate box.

		Your thoughts			How do you believe the majority of clinical ED staff would answer?		
		**Disagree**	**Neutral**	**Agree**	**Disagree**	**Neutral**	**Agree**
**a.**	Improperly cleaned reusable medical equipment contributes to my hospital's rate of Healthcare Acquired Infections (HAIs).	☐	☐	☐	☐	☐	☐
**b.**	Compared to other patient safety issues, HAIs are a significant risk to ED patients.	☐	☐	☐	☐	☐	☐
**c.**	Patients discharged from the ED are at minimal risk from HAIs.	☐	☐	☐	☐	☐	☐
**d.**	The ED does not have a significant impact on my hospital's rate of HAIs.	☐	☐	☐	☐	☐	☐
**e.**	There is close collaboration between infection prevention/control staff and the ED.	☐	☐	☐	☐	☐	☐

GO TO FINAL PAGE ⟶

20) Do staff in your ED speak up to peers or take action when infection control and prevention policies are not followed?

◯ Never ◯ Rarely ◯ Sometimes ◯ Very often ◯ Always

21) Please estimate the combined percentage of ED staff that are new (< 6 months in the ED) and transient members (e.g., rotating medical students, travel pool, float pool), as a percentage of your total ED staff.

◯ 0-19% ◯ 20-39% ◯ 40-59% ◯ 60-79% ◯ ≥80%

22) Please estimate the combined percentage of <u>new</u> (< 6 months in the ED) and <u>transient</u> (e.g., rotating medical students, travel pool, float pool) ED staff members that are <u>correctly trained</u> in infection prevention procedures (to your hospital's policies).

◯ 0-19% ◯ 20-39% ◯ 40-59% ◯ 60-79% ◯ ≥80% ◯ Not applicable

23) Please estimate the percentage of all <u>part-time and full-time</u> staff members dedicated to your ED that are correctly trained in infection prevention procedures (to your hospital's policies), as a percentage of your total ED staff.

◯ 0-19% ◯ 20-39% ◯ 40-59% ◯ 60-79% ◯ ≥80%

<u>Thank you</u>! We welcome your comments and suggestions. Please feel free to attach a note to the survey, or contact us:

Carlos Camargo, MD, DrPH
Massachusetts General Hospital
Phone: 617-724-4069
Fax: 617-724-4050
emnet@partners.org
www.emnet-usa.org

(Optional) **Who completed this survey?** We may want to reach out to you to learn more about your ED. Please complete the fields that you feel comfortable sharing. We will not share your individual contact information.

Name ____________________ Email ____________________

Position (e.g., ED Director) ____________________ Phone ____________________

* Additional information

Question 3: If your ED uses another age to distinguish between children and adults (e.g., age <21 years), and it's difficult to obtain data for age <18 years, please respond according to your ED's cut-off.

Question 4: A pediatric area is any separate physical area where children are seen or sent (e.g., a designated pediatric area of the waiting room, pediatric treatment room/beds). If an area is meant for children but sometimes used for adults in case of overflow, this still counts as a pediatric area.

Question 5: An identified coordinator for pediatric emergency care, or pediatric emergency care coordinator (PECC), is someone who manages pediatric care in the ED and who helps educate other ED staff on pediatric emergency care. PECC roles can vary, and some EDs refer to these individuals with different titles (e.g., Pediatric Champion).

Questions 6 & 7: Telehealth is the use of technology for remote clinical diagnostic and treatment services. This service is usually live audiovisual but may sometimes be only audio (e.g., telephone). Please see the below diagram:

❖ **If your ED *receives* telehealth:**

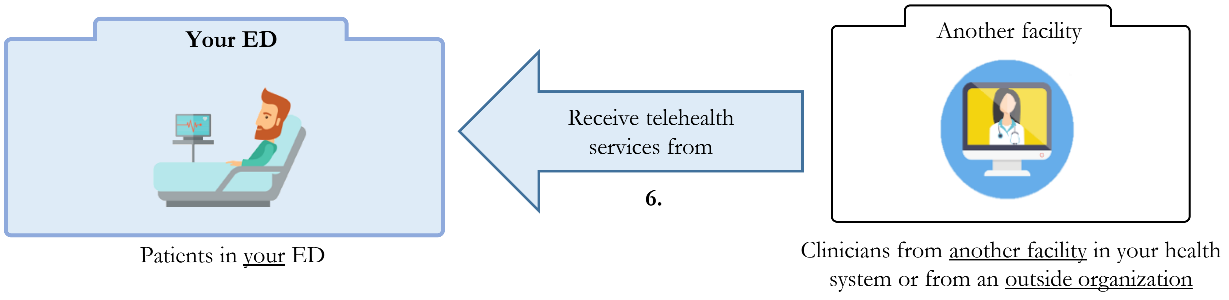

❖ **If your hospital/ED *provides* telehealth *out*:**

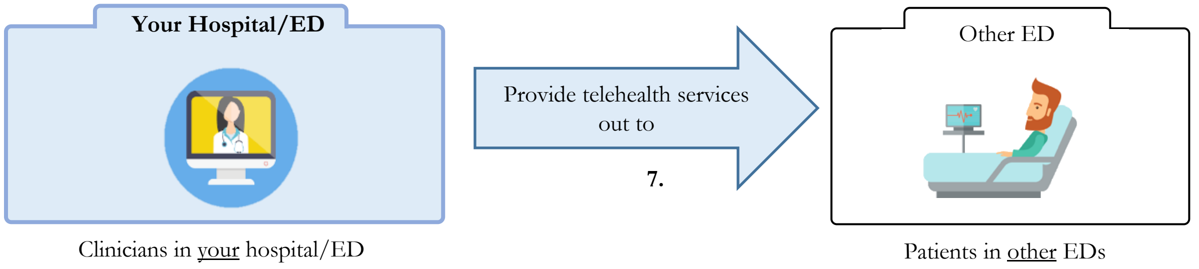

Supplement: Supplementary file 1 [file wjem-26-1781-s001.pdf]
