# Supplementary material for: National Survey on Infection Prevention and Control in United States Emergency Departments
Source: West J Emerg Med. 2025 Nov 26;26(6):1781–9. doi: 10.5811/westjem.46582 (PMC12698160; doi:10.5811/westjem.46582)
Supplement: Supplementary file 2 [file wjem-26-1781-s002.docx]

**SUPPLEMENT B**

In March 2023, one survey question was modified after survey administration began to correct wording that incorrectly asked respondents to calculate the percentage relative to total ED staff rather than the new/transient staff pool. The original question was, “Please estimate the combined percentage of new (< 6 months in the ED) and transient (e.g., rotating medical students, travel pool, float pool) ED staff members that are correctly trained in infection prevention procedures (to your hospital's policies), as a percentage of your total ED staff.” The question was changed to remove the line “as a percentage of your total staff” and to add a “Not applicable” answer choice, for EDs without new or transient staff. Fifty-four EDs had already responded to the survey when the change was made and were re-contacted as applicable to confirm the appropriate response to the revised question.
